# Multilevel Analysis of Psychological Factors in Caregiver Burden Trajectories for Lewy Body Dementia

**DOI:** 10.1093/geroni/igaf122.2527

**Published:** 2025-12-31

**Authors:** Zih-Ling Wang, Yan Su, Oleg Zaslavsky

**Affiliations:** University of Washington School of Nursing, Seattle, Washington, United States; University of Massachusetts Dartmouth, Dartmouth, Massachusetts, United States; University of Washington, Seattle, Washington, United States

## Abstract

This study used secondary data from the parent single-arm pre-post VOCALE LBD (Virtual Online Communities for Aging Life Experiences Lewy Body Dementia) project to examine psychological factors influencing caregiver burden trajectories. The parent study implemented an 8-week asynchronous online intervention specifically designed for Lewy Body Dementia (LBD) caregivers. We analyzed a sample of 39 family/informal caregivers in the intervention group primarily recruited from the Memory and Brain Wellness Center in Seattle, Washington. Participants were predominantly female (69.2%), with a mean age of 67.6 years (SD = 9.6, range 46-91 years). Most caregivers had at least some college education, with 79.5% having completed a bachelor’s degree or higher. Using multilevel regression analysis, we evaluated how caregiver burden changed across three-time points (baseline, post-intervention (8 weeks), and one-month follow-up after intervention end (12 weeks)) and identified key psychological factors influencing these trajectories. Results revealed a significant decrease in caregiver burden during the intervention period (p=.002), with higher self-efficacy associated with steeper decreases (p<.01). However, a significant increase in burden occurred during follow-up (p=.003), particularly associated with increased perceived stress (p<.001). Baseline loneliness (p<.001) and perceived stress (p=.008) significantly predicted higher initial burden levels. Age had differential effects, with older caregivers showing less improvement during the intervention but better maintenance during follow-up. These findings highlight the importance of tailoring interventions based on caregiver characteristics and psychological factors, suggesting that ongoing support after initial intervention may be necessary to maintain improvements in this understudied population.

